# Bayesian reasoning in residents’ preliminary diagnoses

**DOI:** 10.1186/s41235-016-0005-8

**Published:** 2016-09-22

**Authors:** Benjamin Margolin Rottman, Micah T. Prochaska, Roderick Corro Deaño

**Affiliations:** 1 Department of Psychology, University of Pittsburgh, LRDC 726, 3939 O’Hara Street, Pittsburgh, PA 15260 USA; 2 Section of Hospital Medicine, Department of Medicine, University of Chicago, Chicago, IL USA; 3 Division of Cardiovascular Medicine, Department of Medicine, University of Wisconsin School of Medicine and Public Health, Madison, WI USA

**Keywords:** Diagnosis, Clinical reasoning, Base rate neglect, Prevalence

## Abstract

Whether and when humans in general, and physicians in particular, use their beliefs about base rates in Bayesian reasoning tasks is a long-standing question. Unfortunately, previous research on whether doctors use their beliefs about the prevalence of diseases in diagnostic judgments has critical limitations. In this study, we assessed whether residents’ beliefs about the prevalence of a disease are associated with their judgments of the likelihood of the disease in diagnosis, and whether residents’ beliefs about the prevalence of diseases change across the 3 years of residency. Residents were presented with five ambiguous vignettes typical of patients presenting on the inpatient general medicine services. For each vignette, the residents judged the likelihood of five or six possible diagnoses. Afterward, they judged the prevalence within the general medicine services of all the diseases in the vignettes. Most importantly, residents who believed a disease to be more prevalent tended to rate the disease as more likely in the vignette cases, suggesting a rational tendency to incorporate their beliefs about disease prevalence into their diagnostic likelihood judgments. In addition, the residents’ prevalence judgments for each disease were assessed over the 3 years of residency. The precision of the prevalence estimates increased across the 3 years of residency, though the accuracy of the prevalence estimates did not. These results imply that residents do have a rational tendency to use prevalence beliefs for diagnosis, and this finding also contributes to a larger question of whether humans intuitively use base rates for making judgments.

**Electronic supplementary material:**

The online version of this article (doi:10.1186/s41235-016-0005-8) contains supplementary material, which is available to authorized users.

## Significance

Use of Bayes’ rule is a fundamental aspect of rational judgment in probabilistic contexts. Two of the prototypical cases of the use of Bayes’ rule are in the medical domain: prior likelihoods need to be combined with the sensitivity and specificity of a diagnostic test to calculate posttest probabilities, and prevalence beliefs (priors) need to be combined with the knowledge of which diseases cause which signs and symptoms to calculate the likelihood of preliminary diagnoses. The basics of rational probabilistic reasoning using likelihoods and priors are often taught in medical school and in introductory textbooks on diagnosis (Stern, Cifu, and Altkorn, 2010). However, there have been few studies on whether physicians actually use their own beliefs in disease prevalence for diagnosis, and a number of these studies have critical limitations. In the present study, we found that medical residents who had a higher-prevalence belief of a disease rated the disease as more likely in a diagnosis of vignette cases, implying that residents’ diagnoses and prevalence beliefs are connected. This finding should, to some extent, alleviate concern that physicians may be insensitive to base rates when forming preliminary diagnoses. However, this finding emphasizes the importance that physicians have accurate perceptions of prevalence; we found that accuracy was only moderate. Future research should be focused on how prevalence beliefs are learned, whether this learning can be improved, and also how accurate physicians are when using prevalence beliefs for diagnosis.

## Introduction

Diagnostic errors in medicine are major contributors to poor patient outcomes (Gandhi et al., 2006). One of the main causes is physicians’ errors in probabilistic reasoning, such as prematurely settling on a diagnosis (Croskerry, 2002; Graber, Franklin, and Gordon, 2005; Voytovich, Rippey, and Suffredini, 1985). Bayesian reasoning is fundamental to the normative diagnostic process (Ledley and Lusted, 1959; Pauker and Kassirer, 1980). To calculate the posttest likelihood of a disease, Bayes’ rule combines the pretest probability of disease (the prior probability, base rate, or prevalence) and the likelihood ratio (the sensitivity and specificity of the test). The same Bayesian framework also applies when combining the prevalence of a disease with a patient’s symptoms to determine the likelihood of different diagnoses, which was the focus of the present study.

### The effect of prevalence beliefs on diagnosis

Whether, when, and how people use base rates is a subject of long-standing debate (Barbey and Sloman, 2007; Gigerenzer and Hoffrage, 1995; Koehler, 1996). However, most of the experiments providing a basis for this debate on base rate “neglect” or underuse have given participants what resembles an algebra word problem: Participants are provided with the prior probability and likelihood ratio and are expected to come up with Bayes’ rule and apply the equation to the supplied statistics (Agoritsas, Courvoisier, Combescure, Deom, and Perneger, 2011; Casscells, Schoenberger, and Graboys, 1978; Chambers, Mirchel, and Lundergan, 2010; Eddy, 1982; Lyman and Balducci, 1994; Puhan, Steurer, Bachmann, and ter Riet, 2005; Sox, Doctor, Koepsell, and Christakis, 2009; Steurer, Fischer, Bachmann, Koller, and ter Riet 2002). In everyday clinical practice, physicians are not provided with external prevalence estimates. Though they could seek out prevalence estimates from the literature, often they rely on their own beliefs about prevalence estimates, based on either their previous reading of the literature or their experience.

The main question in this study was whether physicians use their beliefs about prevalence for making preliminary diagnoses. This question has existed for a long time within medical communities. Theodore E. Woodward, a famous medical researcher and diagnostician, cautioned students to think of “horses” (common diseases) when hearing hoofbeats (symptoms), not “zebras” (rare diseases). It is possible that physicians spontaneously use their own prevalence beliefs more or less than they use externally provided statistics.

Researchers in two previous studies assessed whether doctors use prevalence beliefs based on their own experience in diagnosis. Unfortunately, these studies have critical limitations that prohibit strong conclusions. In one of the studies (Christensen-Szalanski and Bushyhead, 1981), the researchers found a correlation between physicians’ judgments of the probability of pneumonia given different symptoms (e.g., cough) and the objective predictive value of the symptoms, which has been widely cited as evidence that doctors use base rates (Christensen-Szalanski and Beach, 1982; Koehler, 1996; Medin and Edelson, 1988). However, it is possible that the doctors relied only on their knowledge of which symptoms are more (e.g., crackling sound while breathing) or less (e.g., stomachache) predictive of pneumonia and did not use base rates at all (Kleiter et al., 1997). Additionally, the doctors grossly overestimated the likelihood of pneumonia relative to chest x-ray results, implying that they did not attend to the low base rate of pneumonia.

In another widely cited study, family practitioners judged the likelihood of diagnoses for vignette cases (Weber, Böckenholt, Hilton, and Wallace, 1993). They judged high-prevalence diseases as being more likely than low-prevalence diseases, which is consistent with use of base rates. However, it is also possible that the symptoms were more consistent with the high-prevalence diseases in those vignettes.

In summary, the key studies on whether physicians use the prevalence of diseases when making a diagnosis have strong alternative explanations. Our goal was to test this question with a paradigm that controls for these alternative explanations.

### Origins of prevalence beliefs

Another important question is how physicians develop prevalence beliefs in the first place (Richardson, 1999). Though published prevalence estimates could serve as a general guide, prevalence can vary by geographic location, patient demographics, and clinical setting. There is considerable variability in physicians’ prevalence estimates of diseases (Dolan, Bordley, and Mushlin, 1986). The question addressed here is which factors influence prevalence beliefs among residents.

One likely factor is residents’ own experiences with patients. Each resident treats an idiosyncratic set of patients, which could lead to different prevalence beliefs. Additionally, highly memorable patients may alter subjective judgments of prevalence (Detmer, Fryback, and Gassner, 1978; Lichtenstein, Slovic, Fischhoff, Layman, and Combs, 1978; Tversky and Kahneman, 1973). In the present study, we did not have a way to capture residents’ full experiences, nor could we determine which experiences were most memorable to them. However, we were able to investigate other hypotheses about how experience may influence prevalence beliefs.

Specifically, we hypothesized that residents’ prevalence beliefs may become more precise and accurate across the 3 years of residency. As the residents in the same program gain experience, the law of large numbers tends to make their experiences become more similar, which should increase precision and accuracy. Prevalence estimates may also become more accurate and precise if more experienced residents (measured by residency year) have been exposed to more literature on the true prevalence of a disease.^1^

### Present study

In the present study, we examined the effect of residency year on prevalence judgments, as well as the association between prevalence judgments and diagnostic judgments. We tested whether residents judge a diagnosis as more plausible when they personally believe the disease to have a higher prevalence relative to other residents who believe the disease to have a relatively lower prevalence. The main difference of this approach compared with past research is that we tested whether physicians’ own prevalence beliefs predict their diagnostic judgments, an across-subject, within-disease effect.

We caution that the relationship between prevalence and likelihood of diagnosis is expected to be small. First, as clinical findings accumulate, the prevalence of the diseases normally should become a smaller factor in diagnosis. Even in our short vignettes, there were many clinical findings. Second, as already explained, instead of testing whether residents believe that more prevalent diseases are more likely, we tested whether residents with different beliefs about the prevalence of a single disease make different diagnostic likelihood judgments for that disease. This is a much more subtle effect.

To study the origins of the residents’ prevalence beliefs, we examined the influence of experience on both the accuracy and the precision of prevalence judgments. For precision, we tested whether the standard deviations of the prevalence estimates for a given disease decreased across the 3 years of residency. We assessed the influence of experience on accuracy in two ways. First, we tested whether the absolute deviation between the mean prevalence estimate for each disease and the actual prevalence in the general medicine service at the University of Chicago decreased across the 3 years of residency. Second, we also report the average correlation between an individual resident’s prevalence estimates and the actual prevalence of the diseases as another overall measure of accuracy.

## Methods

### Participants

Residents in the internal medicine residency program at the University of Chicago were recruited by e-mail. Seventy-two of ninety-eight residents participated, and four were dropped from the analyses because they provided repetitive responses, which likely reflected disengagement from the survey. There were 33 year 1 residents (which also included “preliminary” residents completing 1 year of an internal medicine residency before doing a residency in another specialty), 18 year 2, and 17 year 3 residents. The year 1 residents had completed at least 6 months in the residency program before participation.

### Materials

The residents were presented with five vignette cases of hypothetical patients admitted to the general medicine service. The vignettes included pertinent history, signs, symptoms, and vital statistics derived from a physical examination, but not laboratory examination results. The cases were chosen so that, across the 5 cases, there were 24 unique differential diagnoses. (The “differential” is the set of potential diagnoses. Three diagnoses appeared in two vignettes each, resulting in twenty-seven diagnostic judgments.) In order that prevalence beliefs might play a role in diagnosis, the cases were intended to be ambiguous and without a “right” diagnosis at this initial stage; if the symptoms clearly pointed to one diagnosis, there would be no remaining influence of prevalence. Many of the diseases included in the differential diagnosis are fairly common, which was also intended so that the residents’ prevalence beliefs could play a role. Using vignettes of extremely rare diseases would have been more of an exercise in pathophysiological reasoning. Still, including some rare diseases could not be avoided. Table 1 shows the titles of the vignettes and the sets of likely diagnoses. The full vignettes are available in Additional file 1. Four of the vignettes and differential diagnoses are edited versions of case reports used for resident education (Couri and Targonski, 2005; Larochelle and Phillips, 2003; Martinez and Edson, 2004; Schultz, Lassi, and Edson, 2007). We wanted to include a vignette with abdominal pain, a frequent diagnostic challenge, but could not find an appropriate case, and thus wrote it ourselves.

**Table 1:** Five vignettes and differential diagnoses with mean prevalence estimates, actual prevalence rates, and mean diagnostic likelihood estimates

	Mean prevalence estimate	Actual prevalence	Mean diagnostic likelihood estimate	Regression weight log diagnostic likelihood on log prevalence
18-year-old woman with headache and sore throat (Martinez and Edson, 2004)				
Infectious mononucleosis	0.0040	0.0011	0.3469	0.02
Acute viral hepatitis	0.0053	0.0388	0.0544	0.12
Head and neck malignancy	0.0041	0.0004	0.0299	0.00
Primary HIV infection^a^	0.0075	0.0191	0.1193	0.06
*Streptococcus pyogenes* (group A hemolytic)	0.0081	0.0020	0.3415	0.12
19-year-old man with chest pain, fever, & vomiting (Schultz, Lassi, and Edson, 2007)				
Acute myocardial infarction	0.0200	0.0101	0.0250	0.09
Community-acquired pneumonia	0.0806	0.0797	0.1765	0.02
Pulmonary embolus	0.0397	0.0225	0.0928	0.12
Myopericarditis	0.0022	0.0000^b^	0.3954	0.08
Infective endocarditis^a^	0.0088	0.0030	0.2018	0.11
23-year-old woman with diffuse muscle & joint pain (Larochelle and Phillips, 2003)				
Hodgkin’s disease	0.0017	0.0006	0.0692	0.09
Primary HIV infection^a^	0.0081	0.0191	0.0685	0.11
Connective tissue disease (e.g., SLE)^a^	0.0301	0.0431	0.6226	−0.01
Vasculitis	0.0075	0.0088	0.1287	0.00
Demyelinating polyneuropathy	0.0008	0.0002	0.0285	0.07
Hypothyroidism	0.0643	0.0622	0.0528	−0.08
67-year-old woman with abdominal pain				
Abdominal aortic aneurysm	0.0062	0.0077	0.0500	0.13
Appendicitis	0.0057	0.0010	0.1036	0.12
Partial small-bowel obstruction	0.0249	0.0212	0.2543	0.19
Urinary calculus peptic ulcer disease	0.0196	0.0012	0.1174	0.09
Peptic ulcer disease	0.0558	0.0292	0.0860	0.04
Diverticulitis	0.0248	0.0412	0.3584	0.07
61-year-old woman with knee pain and confusion (Couri and Targonski, 2005)				
Transient ischemic attack or stroke	0.0205	0.0096	0.1401	−0.13
Encephalitis	0.0031	0.0016	0.1492	−0.02
Connective tissue disease (e.g., SLE)^a^	0.0301	0.0431	0.0585	0.01
Infective endocarditis^a^	0.0088	0.0030	0.3686	−0.06
Bacteremia	0.0562	0.0273	0.2390	−0.21

*SLE* systemic lupus erythematosus

^a^This diagnosis appeared in more than one vignette

^b^Treated as .0001 for analyses

### Procedure

The study was completed online. Participants first read each of the five vignettes and judged the likelihood (posterior probability) of each diagnosis on the differential. For each vignette. there were five or six likely diagnoses on the differential (Table 1), and there was also a “None of the above” option. Across the six or seven total options, the likelihoods had to sum to 100 %.

After working through all five vignettes, participants reported the prevalence of each of the diagnoses^2^ in Table 1 using the following instructions: “For each diagnosis, please rate how often patients on the general medicine service have that diagnosis. For example, if you choose *x*% for Asthma, that means that *x*% of patients on the general medicine service have asthma.” Participants could choose one of the following 21 options: 0.01 %, 0.02 %, 0.05 %, 0.1 %, 0.2 %, 1 %, 2 %, … 15 %. We used a previously developed technique in which the options above 1 % fell on a linear scale and the options below 1 % fell on a roughly log scale (Woloshin, Schwartz, Byram, Fischhoff, and Welch, 2000). The 21 options were placed on a graphical number line with a magnified scale below 1 %. To make the small percentages easier to understand, we also presented them as fractions (0.01 % = 1 in 10,000).

### Other data sources

For assessing the accuracy of the prevalence estimates, we used a clinical research database that tracks information on patients admitted to the general medicine service at the University of Chicago (Meltzer et al., 2002). Patients’ diagnoses based on International Classification of Diseases, Ninth Revision, codes were obtained from billing reports. A patient was treated as having a diagnosis of interest regardless of whether it was listed as the primary diagnosis or a secondary diagnosis for that hospitalization.

## Findings

### Relationships between prevalence beliefs and diagnostic likelihood judgments

Table 1 gives the mean diagnostic likelihood judgments. The following analyses use log diagnostic likelihood judgments and log prevalence estimates. According to Bayes’ rule, the log diagnostic judgments should equal the log prevalence estimates plus the log-likelihood ratio, which means that log diagnostic judgments and log prevalence estimates should be linearly related (Griffiths and Yuille, 2008).

For each of the 27 diseases, we ran a linear regression to predict the residents’ diagnostic likelihood judgments on the basis of their prevalence estimates. This analysis tests for a between-subjects, within-disease effect. Of the 27 regression weights, 21 were positive, 3 were significant at α = .05, and 2 more were significant at α = .10. Of the six diseases with negative slopes, none were significant at α = .10.

To test whether there was an overall positive effect of the prevalence beliefs on diagnosis likelihood judgments, we ran a one-sample *t* test on the 27 regression weights against 0. On the whole, they were significantly positive [*t*(26) = 2.58, *p* = .015]. A binomial test of 21 of 27 was also significant (*p* = .006).^3^ These findings suggest that believing a disease to be more prevalent is correlated with higher diagnostic likelihood judgments.

### Prevalence estimates

#### Influence of residency year on the precision of prevalence estimates

Precision, in this context, is the closeness of agreement between the residents’ prevalence estimates for a given disease, and it is canonically calculated with the standard deviation (Menditto, Patriarca, and Magnusson, 2006). To determine whether the precision of the prevalence estimates increased across the 3 years of residency, within each year of residency we calculated the standard deviation of the prevalence estimates for each of the 24 unique diseases listed in Table 1. We then compared the standard deviations using an analysis of variance (ANOVA) with year as a continuous predictor and disease as a random factor. We ran three versions of this test using (1) standard deviations, (2) interquartile range of the log prevalence estimates, and (3) coefficient of variation, which is the standard deviation divided by the mean.^4^ As the years increased, the precision of the prevalence estimates increased (standard deviations decreased). This finding was significant in all three analyses: (1) *B* = −0.04, *F*(1,23) = 8.71, *p* < .01, *η*_p_^2^ = 0.12; (2) *B* = −0.07, *F*(1,23) = 4.66, *p* = .04, *η*_p_^2^ = 0.08; and (3) *B* = −0.09, *F*(1,23) = 14.68, *p* < .01, *η*_p_^2^ = 0.11.

#### Influence of residency year on the accuracy of prevalence estimates

In this context, accuracy (otherwise known as “trueness” [Menditto et al., 2006] or the opposite of bias) is the closeness of agreement between the average prevalence estimate of a disease and the actual prevalence of the disease in the general internal medicine service at the University of Chicago. To determine whether the accuracy of the prevalence estimates increased across the 3 years of residency, within each year of residency we calculated the mean of the log prevalence estimates for each of the 24 unique diseases and compared these means with the actual log prevalence.^5^

We took the absolute value of the difference between the mean log prevalence estimates and the actual log prevalence and performed ANOVA with year as a continuous predictor and disease as a random factor. We did not find a significant effect of year [*B* = 0.009, *F*(1,23) = 0.46, *p* = 0.50, *η*_p_^2^ < .001]. This lack of an effect means that the accuracy of the estimates did not systematically change across the 3 years.

#### Correlations between prevalence estimates and actual prevalence

Another way to understand the accuracy of the prevalence estimates of a given resident is to run a correlation between the resident’s log prevalence estimates and the actual log prevalence of the 24 diseases. We then Fisher-transformed these estimates, took the mean, and inverse-transformed the means. The average correlations were virtually identical across the 3 years: year 1 residents *r*_Mean_ = 0.61, year 2 residents *r*_Mean_ = 0.60, and year 3 residents *r*_Mean_ = 0.62. Across all the residents, the weakest correlation was *r* = 0.41 and strongest was *r* = 0.82.

## General discussion

The results of previous research were conflicting as to whether doctors use base rates in diagnosis. The research suggesting that doctors do not use base rates enough have used word problems that do not necessarily reflect typical medical reasoning with one’s own beliefs (Casscells et al., 1978; Eddy, 1982). As argued above in the Introduction section, the published articles suggesting that doctors do use base rates contain serious limitations. In the present study, we tested whether residents who believe a disease to be more prevalent tend to judge the disease as more likely in the differential diagnosis. Though this effect was expected to be subtle, we did find affirmative evidence that residents are sensitive to base rates. These findings are comforting in that residents appear to be “more Bayesian” than we might expect.

One limitation of the present study is that it was impossible to assess whether the residents used their own prevalence beliefs to the right extent. Such an analysis would require assessing the residents’ beliefs about the likelihood of each disease producing the particular constellation of symptoms; these likelihoods are hard to quantify. Simpler cases in which the likelihood ratios can be quantified, such as making pre-post diagnostic judgments before vs. after a diagnostic test, suggest that medical professionals do not use their own pretest beliefs enough. Researchers in another study found that laypeople do not use their own base rate beliefs enough in a Bayesian updating task (Evans, Handley, Over, and Perham, 2002). Though we cannot specify, on the basis of the present study, whether the physicians used their prevalence beliefs as much as they ought to, the study demonstrates that they did use their own prevalence beliefs in a complicated task with many possible diagnoses.

Where do the residents’ prevalence beliefs come from? We found that the prevalence beliefs became more similar (higher precision) over the 3 years of residency but that they did not become more accurate relative to the inpatient general medicine service. This could imply that the driving force in the prevalence estimates was not the residents’ experiences on the general medicine service; if so, presumably they would become more accurate. It is possible that the residents’ prevalence judgments were influenced by other experiences (e.g., outpatient experiences or experiences on other services) or that they were influenced by published prevalence estimates or other socially communicated prevalence beliefs.

In conclusion, this study presents the strongest evidence to date that residents are sensitive to prevalence beliefs when performing a diagnosis. Though their prevalence beliefs are correlated with the actual prevalence in the hospital, the correlations are not extremely high (*r* ^2^ = .37). Thus, helping residents develop accurate prevalence beliefs may improve diagnosis.

## Additional file

Additional file 1: Patient Vignettes. (PDF 38.6 kb)

## Footnotes

1 In the closest prior literature, researchers investigated whether prognostic judgments and initial diagnoses, not prevalence judgments, became more precise and accurate across the 3 years of residency, with mixed results (Dolan et al., 1986; Shapiro, 1977).

2 Participants made 27 diagnostic judgments and then 24 prevalence judgments. The order of the five vignettes, the order of the diagnostic judgments within each vignette, and the order of the prevalence judgments were randomized. The large number of judgments of each type serves as a type of distractor in that it would be hard to remember the diagnostic judgment when later making the prevalence judgment for the same disease.

3 A linear regression with by-subject and by-disease crossed random effects on the intercept and the slope of the prevalence estimates was also significant (*b* = .047, *SE* = .017, *p* = .008).

4 The coefficient of variation is a measure of dispersion of a probability distribution that normalizes for the mean (Woloshin et al., 2000). It is useful in the present context because diseases with higher mean prevalence ratings also tend to have higher standard deviations.

5 For this analysis and the one below, the prevalence of myopericarditis, which was actually zero in the dataset, was treated as having the lowest possible prevalence on the prevalence scale (0.01 %).
