# Supplementary material for: Bayesian reasoning in residents’ preliminary diagnoses
Source: Cogn Res Princ Implic. 2016 Sep 22;1:5. doi: 10.1186/s41235-016-0005-8 (PMC5256430; doi:10.1186/s41235-016-0005-8)
Supplement: Supplementary file 1 — Patient Vignettes. (PDF 38.6 kb) [file 41235_2016_5_MOESM1_ESM.pdf]

Online Supplementary Material: Vignettes

Four of the vignettes were adapted from the sources cited in the titles.

18-Year-Old Woman with Headache and Sore Throat (Martinez & Edson, 2004)

A previously healthy 18-year-old woman was admitted to the general medicine service with a 3-day history of headache, sore throat, and fever. She had developed odynophagia and a fever (temperature, 40° C) with associated chills and rigors. The patient described her headache as global and throbbing constantly without visual changes or photophobia myalgias.

Vital signs on admission included a supine blood pressure level of 73/47 mm HG, a pulse rate of 160/min, and a temperature of 38° C. The patient was in obvious discomfort and preferred to lean her head to the left side; she resisted movement of her head to the right side. Bilateral posterior cervical adenopathy was present, more pronounced on the left. Tenderness to palpation was noted along the entire course of the left sternocleidomastoid. Left tonsillar enlargement was pronounced with marked erythema and no exudate. No abscess was evident. No meningismus was present. Right upper quadrant tenderness was noted without rebound tenderness, guarding, organomegaly, or palpable masses. Findings on the rest of the examination were unremarkable.

Infectious Mononucleosis

Acute Viral Hepatitis

Head and Neck Malignancy

Primary HIV Infection

Streptococcus Pyogenes (Group A Hemolytic)
None of the Above

19-Year-Old Man With Chest Pain, Fever, and Vomiting (Schultz et al., 2007)

A previously healthy man aged 19 years was admitted to the general medicine service with a 5-day history of fever, chills, and night sweats; 2 days of nausea and severe vomiting; and 1 day of acute substernal chest pain that was sharp, radiated to the left shoulder, and was exacerbated both by inspiration and movement. The patient also reported that during the past 2 days his urine had progressively darkened. His medical history was remarkable only for a heart murmur noted in childhood.

The vital signs of the young man, who appeared anxious, were as follows: pulse rate, 120 beats/min; respiratory rate, 24 breaths/min; blood pressure, 110/72 mm Hg; and temperature, 38.4°C. Cardiopulmonary examination revealed a grade II/VI harsh holosystolic murmur heard throughout the precordium with reproducible tenderness to palpation over the sternum. No pericardial friction rubs were audible, and no pathological heart sounds were noted. The patient's lungs were clear to auscultation with normal inspiratory effort. Findings on abdominal examination were within normal limits, and no peripheral stigmata of endocarditis were observed.

Acute Myocardial Infarction

Community-Acquired Pneumonia

Pulmonary Embolus

Myopericarditis

Infective Endocarditis

None of the Above

23-Year-Old Woman with Diffuse Muscle and Joint Pain (Larochelle & Phillips, 2003)

A 23-year-old woman was admitted to the general medicine service with a history of joint pain, swelling of the extremities, and fevers over the course of 10 months. Her symptoms gradually progressed from her hands to involve the major joints of her entire body without any erythema. During the next several months, she experienced generalized stiffness, weakness, mild nausea with vomiting, and an unintentional weight loss of 9 kg. She reported triphasic color change of the fingers when exposed to cold. In recent weeks, the patient had intermittent fevers, with temperatures up to 40° C with no night sweats or any other associated symptoms. Her medical history was remarkable for hypothyroidism secondary to Hashimoto thyroiditis. She used ibuprofen and levothyroxine sodium daily. She denied recent travel and was sexually active.

Physical examination revealed a thin, ill-appearing woman with stiff, slowed movements and difficulty ambulating. Her blood pressure was 90/50 mm Hg with orthostatic changes, and her temperature was elevated at 40° C. Cardiovascular examination identified no murmurs or pericardial friction rub. She had a violaceous rash involving the upper eyelids and a hard oval hyperpigmented patch on her left arm. Nontender palpable cervical lymph nodes and a left axillary node were noted. Various points of tenderness involved the cervical area and all joints of the upper and lower extremities. No effusions were present; however, symmetrical bilateral swelling of the hands and fingers was apparent. Strength was diffusely decreased. Findings on the remainder of the examination were unremarkable.

Hodgkin's Disease

Primary HIV Infection

Connective Tissue Disease (e.g., SLE, etc.)

Vasculitis

Demyelinating Polyneuropathy

Hypothyroidism

None of the Above

A 67 year-old Woman with Abdominal Pain

A 67 year-old woman was admitted to the general medicine service with a 12 hour history of abdominal pain. Initially the pain was described as being located in the central abdomen, but then became more diffuse and severe and was occasionally felt in the left flank and left lower quadrant. The patient also complained of nausea but no vomiting. She has not passed stool since the onset of this pain, but had a normal stool yesterday.

She has a past history of gastroesophageal reflux for which she takes pantoprazole daily, and prior surgery for removal of her left ovary due to a benign cyst 37 years ago. On examination, the patient appears uncomfortable, and her skin is slightly pale and mildly diaphoretic. Her vital signs were the following: temperature of 37° C, pulse of 102, blood pressure of 146/94, and respirations of 18. Her abdomen has decreased bowel sounds, and she is tender to palpation throughout her abdomen with no organomegaly, masses, or rebound. She has no costovertebral tenderness to percussion. The remainder of her physical examination is normal.

Abdominal Aortic Aneurysm

Appendicitis

Partial Small Bowel Obstruction

Urinary Calculus Peptic Ulcer Disease

Peptic Ulcer Disease

Diverticulitis

None of the Above

61-Year-Old Woman with Knee Pain and Confusion (Couri & Targonski, 2005)

A 61-year-old woman was admitted to the general medicine service with acute-onset confusion and bilateral knee pain. Four days before admission, she had been examined by a physician for evaluation of frontal sinus headaches, and a viral syndrome had been diagnosed. Her medical history, including drug allergies, was unremarkable. On physical examination at the hospital admission, the patient was delirious and febrile (temperature, 39.3° C). Her pulse rate was 90/min, blood pressure was 130/70 mm Hg, and a grade 2/6 apical pansystolic cardiac murmur was noted. The patient's knee joints were warm and painful, with small bilateral effusions (more extensive on the right than on the left). A homonymous left-sided visual field deficit was present, and she displayed fluent aphasic speech errors. No other neurologic signs were evident, and the rest of the physical examination findings were unremarkable.

Transient Ischemic Attack or Stroke

Encephalitis

Connective Tissue Disease (e.g., SLE, etc.)

Infective Endocarditis

Bacteremia

None of the Above
